# Health-related quality of life changes of children and adolescents with chronic disease after participation in therapeutic recreation camping program

**DOI:** 10.1186/1477-7525-9-43

**Published:** 2011-06-14

**Authors:** Andrea Békési, Szabolcs Török, Gyöngyi Kökönyei, Ildikó Bokrétás, Annamária Szentes, Gábor Telepóczki, The European KIDSCREEN Group

**Affiliations:** 1 Bátor Tábor Foundation, Budapest, Hungary; 2 Semmelweis University, Institute of Mental Health, Budapest, Hungary; 3 Eötvös Loránd University, Faculty of Education and Psychology, Personality and Health Psychology Department, Budapest, Hungary; 4 National Institute of Child Health, Budapest, Hungary; 5 Semmelweis University, 2nd Department of Pediatrics, Budapest, Hungary; 6 University Clinic Hamburg-Eppendorf, Department of Psychosomatics in Children and Adolescents, Hamburg, Germany

## Abstract

**Background:**

The principals of therapeutic recreation underpin a camping program for children and adolescents living with chronic disease. This study aimed to evaluate the campers' health-related quality of life (HRQoL) before and after the program.

**Method:**

We used the Hungarian version of Kidscreen-52 questionnaire to assess HRQoL. The study sample (n = 115) consisted of children and adolescents aged 10-18 (Mean Age: 13,34; SD: 2,20) collected two months before and two months after camp with the following illnesses: oncology patients (n = 32), diabetes (n = 55) and juvenile immune arthritis (JIA) (n = 28). Repeated measures of multivariate analysis of variance (MANOVA) evaluated pre and post camp changes. We used the Reliable Change Index (RCI) to calculate all the 10 subscales of clinically significant changes.

**Results:**

The Self-perception subscale showed significant positive change from pre camp to post camp with small effect size. Autonomy scores showed time related decline as well as significant time and age group interaction: children under 14 years of age showed a significant moderate effect size decrease on the Autonomy subscale. 32 children (27.8%) showed clinically significant improvement (RCI > 1.96) at least on one subscale. All positive changes were independent of the type of disease, age, gender, and previous camp experience.

**Conclusion:**

The therapeutic recreation camping program had a positive impact on HRQoL of children and adolescents living with cancer, diabetes mellitus and JIA. The experience enhanced their self-perception in all age groups and reduced the autonomy of children under 14 years of age. This study is an innovative use of the KIDSCREEN-52 questionnaire to measure the outcome effectiveness of a psychosocial rehabilitation program and to assess and compare HRQoL of children living with different chronic diseases.

## Background

Chronic conditions are challenging at any age, but this may be especially true during childhood and adolescence. Children with chronic diseases may find themselves restricted by and dependent on parents for practical and emotional support [1]. Overanxious parents may attempt to further restrict their autonomy. The children can be inadvertently excluded from their peers and siblings by concerns about the illness, the fear of pain and the need for continual monitoring [2]. Chronic disease during childhood can also result in more frequent depressive moods and other negative emotions, together with lower satisfaction of life, school performance, self-esteem and self concept [3-8].

Young adults with different chronic conditions are at high risk for psychological symptoms that are aggravated by restricted activity days, unpredictability of symptoms, poor prognosis, the presence of more than one condition, and the presence of hearing and speech problems [9].

Social support plays an important role in the adjustment of children living with chronic diseases [10] especially from classmates and parents [11,12]. Increased social support is associated with better psychosocial adjustment [13] and illness management [14].

General self-esteem seems to be an important reconciling factor between social support and adjustment [15] as well as mediating the relationship between perceived physical appearance and psychological distress (depressive mood) in newly diagnosed pediatric cancer patients [16]. Emotional and social consequences of chronic illness are dependent on perceived physical appearance [17]. During adolescence peer belonging and social acceptance have a high priority. In some diseases - e.g. in JIA or in cancer - visible signs of the disease may lead to negative evaluation and social exclusion.

These results suggest that evaluating and monitoring physical functioning in chronically ill children is important but not sufficient. Living with a chronic condition profoundly affects various aspects of life, and changes the everyday biological, psychological and social functions [18] resulting in impaired HRQoL compared to that of healthy peers.

HRQoL can be an important outcome measure in understanding the impact of chronic illness, and can complement objective clinical measures. HRQoL issues have become more salient and are now a fundamental focus of comprehensive healthcare [19]. The World Health Organisation (WHO) defines quality of life as "an individual's perception of their position in life in the context of the culture and value systems in which they live and in relation to their goals, expectations, values and concerns"[20].

HRQoL can be conceptualized as a multidimensional construct which describes physical, psychological and social functioning. Impaired HRQoL was found in children and adolescents with several chronic conditions, e.g. diabetes, gastrointestinal diseases, cardiac conditions, asthma, obesity, end stage renal disease, psychiatric disorders, cancer, rheumatologic conditions, and cerebral palsy [19]. In a Hungarian study lower scores in the physical functioning component of HRQoL were detected in children 5-18 years of age at a paediatric outpatient cardiology unit compared to healthy controls [21].

The aim of the presented research is to investigate outcome effectiveness of a therapeutic recreation summer camp program (the Bátor Tábor program) for children and adolescents living with cancer, diabetes and JIA regarding the expected changes in HRQoL.

The goals of camping programs for children with chronic illnesses tend to be broad enough to address some of the needs common to many of these children, whilst accommodating individual differences. The range of goals generally includes: providing children with a fun-filled, age appropriate experience where they can acquire activity-related skills; encouraging children to develop a self-sufficient attitude; enhancing self-esteem; providing opportunities for a sense of mastery and efficacy in peer relationships; and helping children learn about their illness either through formal education, or informal peer interaction [22].

These camping programs appear to be effective. An overall conclusion is difficult to state due to variations in design, heterogeneity of subjects, and differences in the definition of camp programs. There has been an increasing demand for accountability and outcome-driven, cost-effective models of care [23], but there is a theoretical deficit on measurable positive outcomes of these camping programs. Camp researchers continue to strive to provide empirical evidence that disease specific camps are beneficial for children with chronic illnesses. There are reports on the effectiveness of such camps on quality of life, besides medical and physiological impacts [24-27].

We tested the hypothesis that the therapeutic recreation camping program of Bátor Tábor has a detectable positive impact on the self-reported physical, psychological, emotional and social aspects of well-being of children and adolescents; which could be detected by a time related positive change in HRQoL. A generic measure of HRQoL was chosen which allows for assessment of adolescents with different diseases.

## Methods

### Design of the study

We designed repeated within-subject measures short-term follow-up. Measures were administered at two separate time periods: 2 months before the camp started ("pre camp"; Time 1); and 2 months after camp ("post camp"; Time 2). The main objective of this prospective pre-post study was to evaluate the impact of the Bátor Tábor program on HRQoL of children and adolescents living with chronic diseases.

### Setting

The Bátor Tábor (Camp of Courage) Foundation was founded in 2001. It is a non-profit organization operating as the Central-Eastern-European Center for Therapeutic Recreation, providing camping programs for children and adolescents living with chronic illnesses. Campers attend the camp program free of charge. The campsite of Bátor Tábor is situated in a pleasant countryside environment in Hungary (for further information see: http://www.batortabor.hu).

Bátor Tábor is a member of the Association of Hole in the Wall Camps launched more than 20 years ago by the Academy Award winning actor, Paul Newman [28]. At present the international camp association has 11 members all over the world [29].

The adventure-based program of the camp addresses the needs of chronically ill children and adolescents on several levels [30], and treats participants with a method called therapeutic recreation [22,31]. There is a sophisticated medical centre on site, which stays in the background away from the activities and the fun [32]. During the day there are opportunities to acquire new skills or improve performance of traditional camping skills such as archery, horseback riding, boating, arts and crafts, team games, and other sports. In the evenings there are "all camp" activities including campfire, beach party, casino, and talent show. Each activity offers a kind of a challenge, often confronting the campers with an unknown situation, which encourages them to step over their real or imagined limitations, and try themselves out in new situations. The completion of the challenges is always accompanied by success and recognition - this process is facilitated by the encouraging and supportive attitude and attention of volunteer staff.

### Participants

The children and adolescents enrolled in this study participated in one of the five separate 8-day sessions of Bátor Tábor in the summer of 2008. Sessions were not mixed in terms of presenting conditions. Campers of the international sessions were not invited to participate.

The medical criterion for children attending the program was the diagnosis of childhood cancer or leukaemia (treatment completed within 5 years), diabetes mellitus or JIA. The referring hospitals from all over the country selected the children for camp with the approval of the medical director of the Bátor Tábor Foundation. There were separate sessions for children (age 7-13) and teenagers (age 13-18) for the oncology and diabetic camps, and a single session for the JIA group (age 10-18). Only the age groups of 10-18 years were involved in the study.

In the summer of 2008 there were 298 Hungarian campers between age 10 to 18 participating in the therapeutic recreation summer program. Thirty nine per cent (n = 115) completed the questionnaire before and after camp. Questionnaires with missing answers were excluded. Fifty nine of the 115 participants had attended previous camps. Table 1 shows the demographic characteristics of the study sample.

**Table 1:** Demographic characteristics of study sample

	Oncology sessions (*n *= 32)	Diabetes sessions (*n *= 55)	JIA session (*n *= 28)	Whole sample (*n *= 115)
Age				
Mean	13.40	13.25	13.43	13.34
SD	2.26	2.18	2.23	2.20
Age group				
Younger than 14	18	31	15	64
Older than 14	14	24	13	51
Gender				
Male	15	26	7	48
Female	17	29	21	67

### Measures

We used the Hungarian version of Kidscreen-52 questionnaire to assess the HRQoL of children and adolescents. The questionnaire consists of 52 items in 10 subscales: Physical Well-being(5 items), Psychological Well-being (6 items), Moods and Emotions (7 items), Self-perception (5 items), Autonomy (5 items), Parent Relations and Home Life (6 items), Financial Resources (3 items), Social Support and Peers (6 items), School Environment (6 items), Social Acceptance (Bullying) (3 items). The questionnaire takes 10-15 minutes to fill in.

KIDSCREEN-52 was developed within an international project 'Screening and Promotion for HRQoL in Children and Adolescents - a European Public Health Perspective' with participation of 13 countries (including Hungary) [7,33]. In all participating countries, including Hungary, after piloting the questionnaire, a representative sample was used to produce normative data. Reliability and validity of KIDSCREEN-52 were proven; Cronbach alphas for the overall sample (for the 13 participated countries) ranged from 0.77 (Social Acceptance, Bullying) to 0.89 (Financial Resources and Psychological Well-being). For convergent and discriminant validity, there was a small to medium level of correlation with two other HRQoL measures, The Pediatric Quality of Life Inventory [34], and the Child Health Illness Profile - Adolescent Edition [35]. The instrument has cross-cultural applicability, and T-values and percentages available for each country stratified by age and gender.

Reliability for Cronbach's Alpha values at T1 ranged between 0.73 for Self-perception and 0.92 for Psychological Well-being and at T2 ranged between 0.74 for Social Acceptance and 0.92 for Financial Resources.

### Procedure

We sent the questionnaires by post and instructed campers to complete the questionnaire by themselves at home 8 weeks before and after the camp session they attended. The introductory letter explained the purpose and nature of the study and stressed that campers' decisions to participate were entirely voluntary, anonymous, and would not affect their possibility of attending the camp. Informed consent was obtained from parents. The request also attached a pre-paid addressed return envelope and a demographic questionnaire that included additional questions about the campers' gender, age and previous camp experience. A reminder letter was sent to all campers within two weeks. The two questionnaires completed by the same person were matched by date of birth, and served as the only identifying information. The data-keeping administrative person completed the registration of questionnaires. The design maintained the anonymity of the participants as the data processors did not have any access to the camp files. The Board of the Bátor Tábor Foundation approved the study design.

We requested the campers to complete the Revised Illness Perception Questionnaire (IPQ-R) [36] as well, but the latter is not a subject of this paper.

### Control group

We invited children and adolescents not participating in Bátor Tábor program in 2008 living with the same chronic diseases and matched for age to participate as the members of a control group. We mailed the KIDSCREEN-52 and IPQ-R questionnaires, demographic questionnaire, return envelope, parental informed consent and an introductory letter explaining the nature of the study and the need for a control group. We asked physicians or nurses from partner hospitals of Bátor Tábor to choose control participants from their own database based on the selection criteria. A physician from the out-patient clinic stamped and signed a letter of approval and encouragement from the hospital. The control group returned 114 pre camp (T1) and 25 post camp (T2) questionnaires from 367 packages sent out by 6 infirmaries. We excluded the data from the control group due to the very low response rate.

### Statistical methods

Statistical analysis used SPSS 15.0, with a significance level of p < 0.05. Descriptive analyses assesed the mean and standard deviation of the ten subscales of the KIDSCREEN-52 questionnaire and the confidence interval (CI: 95%) of means. Cronbach-alphas were calculated as indexes of internal consistency. Chi-square tests detected differences of frequencies among groups, and ANOVA revealed any differences of means of subscales of KIDSCREEN-52 among groups at T1.

Repeated measures of multivariate analysis of variance (MANOVA)) evaluated changes of HRQoL. The dependent variable was the ten subscales of the KIDSCREEN-52 instrument, and gender, age-group, disease-group were the independent variables. The small cell numbers permitted interpretation of only two-way interactions. Morris and DeShon's [37] equation 8 for within subject design compared effects size for T1 and T2 scores using means and standard deviations, and the correlation between the two means.

Reliable Change Index (RCI) calculated significant clinical change [38] by using a subject's pretest (x_1_) and posttest score (x_2_), and the standard error of difference between the two test scores: RC=(x_2_-x_1_)/S_diff. _When RC is greater than 1.96, the posttest score reflects real change.

## Results

### Differences between respondents and non-respondents

We tested the differences between non respondents (those who had not sent back or had not completed the second questionnaire) and respondents (those who had completed the entire questionnaires both times without missing items). Analysis of variance (ANOVA) and chi-square procedures examined the differences between the two groups on the demographic variables of age, gender and education of mother, on disease group, on previous camp experiences and on KIDSCREEN scales. There were no significant differences between the two groups.

### Differences by disease group and by earlier participation in a camping program at T1

We examined the data at T1 by disease group and by previous participation in a camping program. ANOVAs and chi-square test assessed pretest differences between disease groups on age, on gender, on previous camp experience and on KIDSCREEN scales' scores. Table 2 summarizes the means (standard deviations and confidence intervals) of all the KIDSCREEN scales by disease groups at T1 and T2. There was a significant difference among disease groups on two KIDSCREEN scales: Physical Well-being and Self-perception scales (F(2, 112) = 5.372, p < 0.01; F(2, 112) = 3.313, p < 0.05, respectively) at T1. Post hoc analysis indicated that on the Physical Well-being scale the diabetes group (M = 17.84, SD = 3.38) had significantly higher scores than the oncology and the JIA group (M = 15.81, SD = 3.80; M = 15.50, SD = 3.65, respectively), and the diabetes group had higher scores (M = 19.58; SD = 3.98) on the Self-perception scale than the JIA group (M = 17.07, SD = 4.96).

**Table 2:** Mean scores (Standard Deviation) [Confidence Interval 95%] of KIDSCREEN-52 instrument at T1 and T2 by disease group and for the whole sample

	Pre camp				Post camp			
**Subscales** **of the KIDSCREEN-52 instrument**	**Diabetes** **Group** *n *= 55	**Oncology group** *n *= 32	**JIA** **Group** *n *= 28	**Whole** **Sample** *n *= 115	**Diabetes** **Group** *n *= 55	**Oncology group** *n *= 32	**JIA** **Group** *n *= 28	**Whole** **Sample** *n *= 115

Physical well-being	17.84 (3.38) [16.92-18.75]	15.81 (3.80) [14.44-17.18]	15.50 (3.65) [14.08-16.92]	16.70 (3.70) [16.02-17.38]	17.53 (3.04) [16.71-18.35]	16.66 (3.29) [15.47-17.85]	14.89 (3.96) [13.35-16.43]	16.64 (3.48) [16.00-17.28]

Psychological well-being	24.58 (5.01) [23.23-25.93]	23.97 (4.62) [22.30-25.64]	23.75 (5.63) [21.57-25.93]	24.21 (5.04) [23.29-25.13]	24.91 (4.51) [23.69-26.13]	24.81 (3.86) [23.42-26.20]	23.64 (5.19) [21.63-25.65]	24.57 (4.51) [23.75-25.39]

Moods & Emotions	29.73(4.17) [28.60-30.86]	28.53 (5.84) [26.42-30.64]	28.71 (5.99) [26.39-31.03]	29.15 (5.13) [28.21-30.09]	29.56 (4.79) [28.27-30.85]	29.84 (3.99) [28.40-31.28]	29.04 (4.80) [27.18-30.90]	29.51 (4.55) [28.68-30.34]

Self-Perception	19.58 (3.98) [18.50-20.66]	18.91 (3.90) [17.50-20.32]	17.07 (4.96) [15.15-18.99]	18.78 (4.30) [17.99-19.57]	20.29 (3.79) [19.27-21.31]	19.66 (3.39) [18.44-20.88]	17.75 (4.83) [15.88-19.62]	19.50 (4.07) [18.76-20.24]

Autonomy	20.13 (4.50) [18.91-21.35]	19.88 (4.48) [18.26-21.50]	19.14 (3.80) [17,67-20,61]	19.82 (4.31) [19.03-20.61]	19.76 (3.93) [18.70-20.82]	18.50 (4.44) [16.90-20.10]	17.64 (4.47) [15.91-19.37]	18.90 (4.27) [18.12-19.68]

Parent Relations & Home Life	24.69 (5.01) [23.34-26.04]	24.69 (5.40) [22.74-26.64]	23.25 (4.99) [21.32-25.18]	24.34 (5.11) [23.41-25.27]	25.65 (4.43) [24.45-26.85]	24.66 (4.63) [22.99-26.33]	23.07 (5.65) [20.88-25.26]	24.75 (4.88) [23.86-25.64]

Financial Rresources	10.93 (3.80) [9.90-11.96]	11.44 (3.23) [10.28-12.60]	10.96 (3.84) [9.47-12.45]	11.08 (3.64) [10.41-11.75]	11.82 (3.09) [10.98-12.66]	11.16 (3.19) [10.01-12.31]	10.50 (3.85) [9.01-11.99]	11.31 (3.33) [10.70-11.92]

Social Support & Peers	24.80 (5.07) [23.43-26.17]	22.69 (5.47) [20.72-24.66]	24.36 (5.09) [22.39-26.33]	24.10 (5.22) [23.15-25.05]	25.36 (4.42) [24.17-26.55]	21.53 (5.74) [19.46-23.60]	23.00 (5.75) [20.77-25.23]	23.72 (5.37) [22.74-24.70]

School environment	21.15 (6.00) [19.53-22.77]	21.13 (5.17) [19.27-22.99]	20.54 (5.71) [18.33-22.75]	20.99 (5.67) [19.95-22.03]	22.13 (5.47) [20.65-23.61]	21.72 (4.22) [20.20-23.24]	21.29 (4.84) [19.41-23.17]	21.81 (4.97) [20.90-22.72]

Social Acceptance (Bullying)	13.91 (1.96) [13.38-14.44]	13.53 (2.26) [12.72-14.34]	13.29 (2.54) [12.31-14.27]	13.65 (2.19) [13.25-14.05]	14.20 (1.65) [13.75-14.65]	13.47 (1.98) [12.76-14.18]	13.39 (1.97) [12.63-14.15]	13.80 (1.85) [13.46-14.14]

### Changes in HRQoL

Repeated measures of mixed factorial MANOVA examined any changes in children's HRQoL - 2 (gender) × 2 age group (younger than 14 years old, older than 14 years old) × 3 (disease group: diabetes, oncology, JIA) × 2 (Time). When multivariate analysis indicated significant results the source of these effects were identified through univariate analysis. Only two-way interactions were interpreted.

There was a significant main effect for Time, F(10, 94) = 3.284, p < 0.01. Univariate analysis found Self-perception scores to change by Time, F(1, 103) = 6.343, p < 0.05. The scores increased slightly from Time 1 (M = 18.78, SD = 4.30, CI: 17.99-19.57) to Time 2 (M = 19.50, SD = 4.07, CI: 18.76-20.24). Autonomy scores changed by Time, F (1, 103) = 6.521, p < 0.05; these scores decreased from Time 1 (M = 19.82, SD = 4.31, CI: 19.03-20.61) to Time 2 (M = 18.90, SD = 4.27, CI: 18.12-19.68). School Environment scores also changed by Time, F(1, 103) = 6.554, p < 0.05; scores of this scale increased from Time 1 (M = 20.99, SD = 5.67, CI: 19.95-22.03) to Time 2 (21.81, SD = 4.97, CI: 20.90-22.72). Effect sizes were small for Self-perception and for Autonomy scales (0.22 and 0.23), respectively, and effect size for School Environment was negligible (0.16)(Table 2).

Significant Time × Gender interaction for Psychological Well-being scale scores was found, F(1, 103) = 4.054 p < 0.05; male scores increased from Time 1 (M = 24.08, SD = 4.95, CI:22.64-25.52) to Time 2 (M = 25.02, SD = 4.08, CI: 23.84-26.20), and effect size was small (0.21). Female scores did not change from Time 1 (M = 24.30, SD = 5.13, CI: 23.07-25.53) to Time 2 (24.25, SD = 4.80, CI: 23.10-25.40).

Significant Time × Age group interaction was found for Autonomy, Parent Relation and School Environment scales' scores (F(1, 103) = 6.061 p < 0.05; F(1, 103) = 5.414, p < 0.05; F(1, 103) = 4.520, p < 0.05, respectively).

Autonomy scores for children under 14 years old (N = 64) decreased from Time 1 (M = 20.64, SD = 3.64, CI: 19.75-21.53) to Time 2 (18.78, SD = 4.18, CI: 17.76-19.80), and effect size was moderate (0.52). Autonomy scores for children older than 14 years old (N = 51) did not change from Time 1 (M = 18.78, SD = 4.88, CI: 17.41-20.15) to Time 2 (19.04; SD = 4.42, CI: 17.80-20.28).

Parent Relation scores for younger children did not change from Time 1 (M = 25.66, SD = 3.93, CI: 24.70-26.62) to Time 2 (25.61, SD = 4.10, CI: 24.61-26.61), while for older children scores increased from Time 1 (M = 22.69, SD = 5.92, CI: 21.02-24.36) to Time 2 (M = 23.67, SD = 5.55, CI: 22.11-25.23), and effect size was small (0.20).

School Environment scores for younger children from Time 1 (M = 22.02, SD = 5.42, CI: 20.69-23.35) did not change to Time 2 (22.20; SD = 4.91, CI: 21.00-23.40), while for older children scores increased from Time 1 (M = 19.71; SD = 5.75, CI: 18.09-21.33) to Time 2 (M = 21.31; SD = 5.05, CI: 19.89-22.73), and effect size was small (0.26).

### Clinically significant changes - Reliable Change Index

Reliable Change Index (RCI) [38] was calculated for all the 10 subscales in order to detect clinically significant changes in our study. According to our results 32 children (27.8%) showed clinically significant improvement (RC > 1.96) on at least one subscale of KIDSCREEN-52 instrument. Specifically, we found that scores of 17 children increased on one scale, scores of 10 children improved on two scales, 3 children showed improvement on three subscales, scores of 1 child increased on four scales and scores of 1 child on six scales.

## Discussion

We can conclude in general from comparison of the pre camp and post camp scores that participation in the therapeutic recreation camping program of Bátor Tábor has significant positive effects. The repeated measure mixed factorial MANOVA showed a significant main effect for time. Clinically significant changes were detected in a remarkable proportion (27.8%) of children and adolescents on at least one subscale of the KIDSCREEN-52 instrument. These positive changes were independent of the type of disease, age, gender, and previous camp experience. This study demonstrated that summer camps in Bátor Tábor for children and adolescents with chronic illnesses could be beneficial in helping to improve campers' HRQoL. In the following sections we will discuss our findings regarding significant changes on subscales separately and consecutively.

### Self-perception

Self-perception turned out to be one of the subscales of the KIDSCREEN-52 instrument which showed significant positive change from T1 to T2, although the effect size was found to be small. These results indicate that campers benefit from the therapeutic recreation program in a very important area. Adolescents experiencing cancer often have low self-concept [39], whereas positive self-concept is generally associated with increased coping abilities [40]. Therefore reinforcement of self-perception, self-esteem and self-efficacy as part of an overall physical and psychosocial rehabilitation is a major goal of therapeutic recreation programs [30] as well as adventure therapy [41]. These current results correspond to the previous research at Bátor Tábor demonstrating significant positive changes in the self-esteem and self-efficacy of adolescents living with diabetes and cancer [42].

### Autonomy

Time related decline as well as significant time and age group interaction was found regarding Autonomy scores: children below 14 years of age showed significant moderate effect size decrease on the Autonomy subscale whereas children older than 14 did not show any change. Autonomy refers to the child's or adolescent's freedom of choice, self-sufficiency and independence, the extent to which the child/adolescent feels able to shape his/her own life as well as being able to make decisions about day-to-day activities [7]. No publications were found on longitudinal changes in autonomy as an outcome result of therapeutic interventions. Studies assessing autonomy as part of the HRQoL of survivors of childhood cancer, in general, reported inconsistent results. The HRQoL of child and adolescent retinoblastoma survivors were found to be higher on Autonomy subscales compared to the population based reference group of the country in the Netherlands [43]. However, there are publications suggesting just the opposite - assuming that living with a chronic condition is associated with increased dependency and lower level of autonomy [44,45].

Wysocki et al found that the group of children with excessive self-care autonomy demonstrated less favorable treatment adherence, diabetes knowledge, hospitalization rates, and glycemic control. They emphasize that children's self-care autonomy has to be balanced with their psychological maturity, and suggest that families who succeed in maintaining parental involvement in diabetes management may have better outcomes [46].

In our study the changes in Autonomy scores in the lower age group (below 14 years) are considered noteworthy. Coping with the stress of having a chronic illness, understanding the disease itself, as well as dealing with its possible outcomes and long-term sequelae, and being involved in the tough course of decision making, reinforces the early maturation of these children and may be connected to enhanced level of autonomy.

We interpret our result in keeping with one of our camper's words on his experiences at camp: "Bátor Tábor is a place where all the positive energies previously having been taken away by the disease are recharged." The environment providing continuous care and supervision by trained counselors, as well as the challenging programs and fun and joy experienced together with peers, seems to make up for what these chronically sick children (especially in the lower age group) have missed out on due to their disease. The decrease in autonomy especially in the younger age-group can be considered as a beneficial outcome of the therapeutic recreation program and noteworthy enough for further research in the future.

### Gender and age-group differences

Further statistical evaluation revealed a significant time and gender interaction in our study. Psychological Well-being showed improvement in boys, whereas Parental Relationships and School Environment subscales showed positive changes in adolescents over 14 years of age.

The School Environment subscale explores an evaluation of being at school and a perception of cognitive capacity. Successful school experiences contribute to feeling of normality among adolescents suffering from chronic conditions [47].

Quality of interaction with parents in our study was an outcome measure, and we found a significant improvement among adolescents over 14 years of age. This result can be important in light of the consistent body of evidence supporting that the parent-child communication or quality of interaction is associated with disease outcomes, e.g. in diabetes [48,49]. Future studies should focus on the prospective relationship between improvement of quality of parent-child interaction and illness management.

### Comparison of disease groups

Further evaluation of pre camp questionnaires (T1 data) enabled us to get information on the differences between the examined disease groups in relation to HRQoL. Children with diabetes were found to show higher scores on the Physical Well-being subscale compared to that of children with cancer or JIA. As compared to the JIA group the Self-perception of children with diabetes also proved to be higher. Our explanation of these findings is related to the fact that chronically sick children and adolescents are challenged in different ways in their everyday life due to the divergent characteristics of symptoms and the course of the disease. After the implementation of proper treatment and education on leading their life with continuous control of their carbohydrate metabolism and blood sugar level children with diabetes are more or less free of symptoms and pain whereas children with cancer experience a great deal of pain and suffering due to the symptoms of the disease as well as the treatment based on chemotherapy, irradiation and surgery. Children with JIA suffer a remitting and flaring course of considerable pain and fatigue, stiffness and disability [50]. These characteristic differences correlate well with our data.

### Limitations of the study

Several limitations of the study should be noted. The inclusion of a control group would have considerably strengthened casual inferences. The questionnaires were filled in an uncontrolled environment (campers' home) that varied from child to child and is a potential bias in our results. In addition, neither the time of diagnosis nor the age at diagnosis were considered.

There are studies demonstrating that HRQoL is dependent on the progress of the illness. In a prospective study differences between adolescent cancer patients and healthy controls gradually disappeared and then reversed resulting in the cancer group reporting significantly better HRQoL and lower levels of anxiety and depression than the reference group a year and a half from diagnosis [51].

There was lack of information on family socio-demographic characteristics, and life events (e.g. loss of a significant person), which were consequently not controlled for between the pre and post camp administration of the questionnaires in our study.

Another limitation of the study is that the design cannot account for possible specific components of therapeutic recreation contributing to the positive outcome. Nevertheless, the results show notable psychological benefit associated with camp participation, despite the absence of specific and structured psychological interventions.

## Conclusions

The present study assessed the changes of HRQoL of children and adolescents with chronic illnesses as a result of participation in a therapeutic recreation camping program. The program proved to have a positive impact on the HRQoL of children and adolescents living with cancer, diabetes and JIA, especially enhancing their self-perception and reducing the autonomy of children less then 14 years of age.

The novelties of this study are the use of the KIDSCREEN-52 questionnaire to measure outcome effectiveness of a psychosocial rehabilitation program and assessing and comparing the HRQoL of children living with cancer (excluding diabetes mellitus and JIA).

In our experience the KIDSCREEN-52 questionnaire proved to be an appropriate tool to measure HRQoL of children and adolescents living with cancer, diabetes and JIA, as well as measuring effectiveness of a therapeutic recreational camping program.

The comparison of HRQoL among a larger group of children and adolescents living with different chronic conditions would provide further data useful for informing future program designs. The aim is to achieve long-lasting positive changes in the HRQoL of chronically ill children and adolescents through therapeutic recreation rehabilitation programs precisely adjusted to the different needs arising from the varying psychosocial demands caused by dissimilar diseases.

## Future directions

This report and our previous study [42] prove that participation in the camping program in Bátor Tábor has an impact on self-evaluation. Further research is required to clarify which component of self-evaluation changes are due to therapeutic recreation (e.g. self-acceptance, contingent self-esteem).

There was a positive change found in parent-child relationship. Adolescents' HRQoL is influenced by the broader social context (family, peers) and extensive research shows a complex and bidirectional relationship among social factors (e.g. social support, satisfaction with support) and chronic conditions in youth. Changes in self-esteem and relationship with parents may be related to each other. A more positive self-schema may lead to improvement in relationships, whereas better relationships may strengthen self-esteem. Detection of this dynamic relationship between psychological and social variables requires more follow-up studies.

There is a plan to incorporate the presentation of the results of the study into the training of camp volunteer staff in order to raise their awareness of how HRQoL of chronically sick children can be influenced by therapeutic recreation as well as to call their attention to the importance of their role in the therapy.

## List of abbreviations used

ANOVA: Analysis of Variance; CI: Confidence Interval; HRQoL: Health-Related Quality of Life; IPQ-R: Revised Illness Perception Questionnaire; JIA: Juvenile Immune Arthritis (rheumatoid arthritis); M: Mean; MANOVA: Multivariate Analysis of Variance; RCI: Reliable Change Index; SD: Standard Deviation.

## Competing interests

The authors declare that they have no competing interests.

## Authors' contributions

AB, ST, GK, IB, AS and GT designed the study protocol. AB, IB and GT organized and carried out the acquisition of data. GK performed the statistical analysis. AB and ST made major contributions to interpretation of data. GK, IB and AS have been involved in revising the manuscript. All authors read and approved the final manuscript.
